# An Autopsy Study of Maternal Mortality in Mozambique: The Contribution of Infectious Diseases

**DOI:** 10.1371/journal.pmed.0050044

**Published:** 2008-02-19

**Authors:** Clara Menéndez, Cleofé Romagosa, Mamudo R Ismail, Carla Carrilho, Francisco Saute, Nafissa Osman, Fernanda Machungo, Azucena Bardaji, Llorenç Quintó, Alfredo Mayor, Denise Naniche, Carlota Dobaño, Pedro L Alonso, Jaume Ordi

**Affiliations:** 1 Barcelona Center for International Health Research (CRESIB) and Department of Pathology, Hospital Clinic, Institut d'Investigaçions Biomediques August Pi i Sunyer (IDIBAPS), Universitat de Barcelona, Spain; 2 Centro de Investigaçao em Saude de Manhiça (CISM), Maputo, Mozambique; 3 Department of Pathology, Maputo Central Hospital, Maputo, Mozambique; 4 National Malaria Control Program, Ministry of Health, Mozambique; 5 Department of Gynaecology, Maputo Central Hospital, Universidad Eduardo Mondlane, Maputo, Mozambique; National Institute of Child Health and Development, United States of America

## Abstract

**Background:**

Maternal mortality is a major health problem concentrated in resource-poor regions. Accurate data on its causes using rigorous methods is lacking, but is essential to guide policy-makers and health professionals to reduce this intolerable burden. The aim of this study was to accurately describe the causes of maternal death in order to contribute to its reduction, in one of the regions of the world with the highest maternal mortality ratios.

**Methods and Findings:**

We conducted a prospective study between October 2002 and December 2004 on the causes of maternal death in a tertiary-level referral hospital in Maputo, Mozambique, using complete autopsies with histological examination. HIV detection was done by virologic and serologic tests, and malaria was diagnosed by histological and parasitological examination. During 26 mo there were 179 maternal deaths, of which 139 (77.6%) had a complete autopsy and formed the basis of this analysis. Of those with test results, 65 women (52.8%) were HIV-positive. Obstetric complications accounted for 38.2% of deaths; haemorrhage was the most frequent cause (16.6%). Nonobstetric conditions accounted for 56.1% of deaths; HIV/AIDS, pyogenic bronchopneumonia, severe malaria, and pyogenic meningitis were the most common causes (12.9%, 12.2%, 10.1% and 7.2% respectively). Mycobacterial infection was found in 12 (8.6%) maternal deaths.

**Conclusions:**

In this tertiary hospital in Mozambique, infectious diseases accounted for at least half of all maternal deaths, even though effective treatment is available for the four leading causes, HIV/AIDS, pyogenic bronchopneumonia, severe malaria, and pyogenic meningitis. These observations highlight the need to implement effective and available prevention tools, such as intermittent preventive treatment and insecticide-treated bed-nets for malaria, antiretroviral drugs for HIV/AIDS, or vaccines and effective antibiotics for pneumococcal and meningococcal diseases. Deaths due to obstetric causes represent a failure of health-care systems and require urgent improvement.

## Abstract 2

Clara Menendez and colleagues analyze 139 complete autopsies following maternal deaths in Maputo, Mozambique and find a predominance of infectious and preventable causes.

## Editors' Summary

**Background.:**

Every year, half a million women—many of them living in developing countries—die during pregnancy or childbirth or within a few weeks of delivery. (The term “maternal deaths” is used to designate such deaths.) For women living in sub-Saharan Africa, the situation is particularly grim. Half of all maternal deaths occur in this region. The maternal mortality ratio (MMR)—the number of maternal deaths per 100,000 live births—in sub-Saharan Africa is nearly 1,000; in industrialized countries it is 8. The lifetime risk of maternal death in sub-Saharan Africa is 1 in 22; in industrialized countries it is 1 in 8,000. Faced with the magnitude of the global maternal death toll, in September 2000 the United Nations pledged, as its fifth Millennium Development Goal, that the global MMR would be reduced to a quarter of its 1990 level by 2015. Currently, it seems unlikely that this target will be met. Between 1990 and 2005 global maternal deaths decreased by only 1% per annum. In sub-Saharan Africa the annual reduction was even less—0.1% per annum.

**Why Was This Study Done?:**

One reason for this slow progress is that public-health professionals in developing countries rarely have accurate data about the causes of maternal death, information that they need to guide their efforts to reduce these deaths. A detailed examination of the body after death (a medical autopsy) is the only sure way to ascertain the causes of maternal death, but in most developing countries, clinical records and verbal autopsies (asking relatives about the circumstances of the mother's death) are the main sources of these data and neither source is optimally accurate. The currently available information indicates that birth (obstetric) complications are the most frequent causes of maternal death in developing countries, in particular, hemorrhage (uncontrollable bleeding) after the baby is born. However, little is known about the impact of the HIV/AIDS epidemic (which is worst in Sub-Saharan Africa), malaria, or other infectious diseases on maternal deaths. In this study, the researchers use complete autopsies to determine the causes of maternal death in the Maputo Central Hospital, Mozambique, a tertiary-level hospital to which women with high-risk pregnancies are referred for specialized care.

**What Did the Researchers Do and Find?:**

Between October 2002 and December 2004, there were 179 maternal deaths in the Maputo Central Hospital and 31,135 live births, corresponding to a ratio of 874 maternal deaths per 100,000 live births. (Because the hospital was a referral center, this ratio would not be expected to reflect the actual MMR for the general population of the Maputo area.) Complete autopsies were done on 139 of the women, HIV infection was measured using standard tests, and malaria was diagnosed by looking for parasites and malaria-associated changes in postmortem samples. Of these 139 women, just over one-third died because of obstetric complications; hemorrhage was the most common cause of death (one in six maternal deaths). The commonest nonobstetric causes of maternal death were HIV/AIDS- related conditions, including infections and cancers (about 1 in 8 maternal deaths; about half the women in the study were HIV positive). Other common causes were pyogenic (pus-forming) bacterial infections of the lungs and brain, and malaria. Together, these infectious diseases accounted for nearly half of the maternal deaths.

**What Do These Findings Mean?:**

These findings indicate that infectious diseases account for a large proportion of maternal deaths at Maputo Central Hospital and identify which obstetric complications are responsible for most maternal deaths in this setting. They may not, however, accurately reflect the causes of maternal death elsewhere in Mozambique. For example, maternal deaths from some obstetric complications may be over-represented in this study because women at risk of these complications would have been referred to this hospital. Conversely, the proportion of women dying from hemorrhage may be higher in the community because this complication usually happens shortly after birth, leaving little time for women to reach a hospital for treatment. Nevertheless, these findings suggest that the implementation of effective measures to prevent and treat HIV/AIDS, malaria, and infections with pyogenic bacteria, together with improvements in health services for obstetric complications, should greatly reduce the maternal death toll in Mozambique and perhaps in other countries in sub-Saharan Africa.

**Additional Information.:**

Please access these Web sites via the online version of this summary at http://dx.doi.org/10.1371/journal.pmed.0050044.

- UNICEF (the United Nations Children's Fund) provides information on maternal mortality including the WHO/UNICEF/UNFPA/The World Bank) estimates of maternal mortality for 2005 by country, and an article on maternal mortality in the Cabo Delgado province of Mozambique
- More information on the WHO/UNICEF/UNFPA/The World Bank estimates of maternal mortality in 2005
- The UK Department for International Development provides information about Millenium Development Goal 5: the improvement of maternal health
- The US Centers for Disease Control and Prevention has a page on pregnancy-related deaths (in English and Spanish)
- The Partnership for Maternal, Newborn and Child Health provides information on maternal deaths (in English, Spanish, French, Russian, Arabic, and Chinese)
- The United Nations Development Fund for Women (UNIFEM) focuses on improving conditions for women worldwide, especially those in poverty

## Introduction

Every year approximately 250,000 African women die during pregnancy, delivery, or the puerperium, but we know very little of the causes of these deaths. In Africa maternal mortality ratios (number of maternal deaths per 100,000 live births) are more than 100 times higher than those in the developed world [1], and the international community has included the reduction of maternal mortality by three-quarters by 2015 as one of the targets of the Millennium Development Goals [2]. However, efforts to reduce maternal mortality in Africa are not being driven by evidence. In developing countries the main source of information on the causes of maternal death is clinical records and verbal autopsies [2–10]. Both sources have substantial limitations due to discrepancies between the clinically presumed and the actual cause of death [11]. Information provided by medical autopsies has played an important role in increasing the accuracy of cause-of-death reports and improving clinical practice in the developed world [11,12]. Autopsies may also provide important data on the causes of maternal death, an essential component to reducing maternal mortality and to directing public health efforts.

According to available information, obstetric complications are the most frequent causes of maternal mortality in developing countries [2,6–8,13]. In the last two decades the HIV/AIDS epidemic has become a major health problem in sub-Saharan Africa [14], but its impact on maternal mortality has been poorly investigated [15]. Similarly, little is known on the possible impact of severe malaria [16,17] and other infectious diseases. Recent evidence suggests that HIV infection increases the risk of malaria among pregnant women [18], but the possible effect of this interaction on mortality has not been evaluated.

In order to accurately determine the causes of maternal death in a tertiary-level referral hospital in a country in sub-Saharan Africa, we conducted a prospective, descriptive study in Maputo, Mozambique, that included complete autopsies.

## Methods

### Study Area

The study was conducted at the Maputo Central Hospital, between October 2002 and December 2004. The Maputo Central Hospital is a government-funded tertiary care facility, and serves as the referral centre for other hospitals in Southern Mozambique. Malaria transmission in the Maputo urban and suburban areas is low [19], while the surrounding rural areas have moderate and stable transmission. The reported HIV seroprevalence in pregnant women for 2004 was 16.2% as the average for the country, and 20.7% for the Maputo area (unpublished data, Ministry of Health, Mozambique, 2004).

### Study Design

This was a prospective, descriptive study that included all consecutive deaths fulfilling the standard definition of the World Health Organization (WHO) for a maternal death. We included all women dying during pregnancy or within 42 d of completion of a pregnancy, irrespective of the cause of death, and for whom the family had given verbal (oral) informed consent. A standardized questionnaire to collect demographic, clinical, and obstetric data was administered by trained health personnel after a review of the medical records. All births occurring at the hospital during the study period were reviewed to estimate the ratio of maternal deaths to live births among mothers who gave birth at the hospital. The study protocol was approved by the National Mozambican Ethics Committee and the Hospital Clinic of Barcelona Ethics Review Committee.

### Autopsy and Diagnosis

Maternal deaths underwent a complete dissection with macroscopic evaluation of each organ by a pathologist (CC, MRI, or CR) using a standardized macroscopic protocol. Samples of all grossly identified lesions and of all viscera were collected from each woman for histological study. A blood sample (100 μl) was obtained from the inferior vena cava and stored on filter paper.

The final diagnoses were established by two pathologists (CR, JO) after reviewing the histological slides and the macroscopic and clinical protocols. Clinical and necropsy diagnoses were grouped into different categories according to the International Classification of Disease, tenth revision (ICD-10). HIV/AIDS-related diseases included all cases with opportunistic infections or other conditions included in the CDC revised criteria [20]. When more than one pathological diagnosis was identified, the different diagnoses were classified as: (a) main disease causing the death; (b) final event directly causing the death in a patient with a different main diagnosis (e.g., aspiration pneumonia in a woman with eclampsia causing a cerebral haemorrhage); (c) secondary lesions possibly contributing to the death (e.g., past malaria diagnosed because of macrophages with haemozoin in liver and spleen in a patient dying of meningitis); and (d) associated incidental lesions not contributing to the death (e.g., schistosomiasis). The study was mainly focussed in main diseases causing the death (category a).

### Laboratory Methods

Tissue specimens were fixed in 10% buffered formalin for 2–15 d and embedded into paraffin wax using standard procedures. For each sampled tissue, 4 μm sections were stained with haematoxylin and eosin (H&E). Ancillary histochemical and immunohistochemical stains were performed to confirm or exclude specific lesions suspected on the H&E stains.

Detection of integrated HIV provirus was determined by qualitative DNA PCR using the standard Amplicor HIV-1 kit (Roche) on blood collected onto filter paper. In 46 women, HIV status was assessed prior to death using the rapid test Determine HIV (Abbot Laboratories), and positive results were confirmed with Unigold HIV (Trinity Biotech).

Malaria parasitaemia was assessed in 61 women prior to death on thick and thin air-dried blood films, stained with Giemsa. A histologic evaluation of malarial pigment was done with light microscopy under polarized light [21,22].

### Definitions

Severe malaria was defined as: (a) abundant sequestered parasites in the central nervous system (CNS); (b) abundant sequestered parasites in tissues other than the CNS and massive haemozoin deposits in macrophages in the sinusoids of the liver and spleen (in the absence of other causes of death); and (c) clinical symptoms of severe malaria and positive parasitaemias prior to death, with massive haemozoin deposits in the sinusoids of the liver and spleen associated with indirect signs of severe malaria in the autopsy (ring haemorrhages in the CNS, intravascular disseminated coagulation, or pulmonary oedema), independent of the presence or absence of parasites in the autopsy study. All other cases with parasites in the autopsy or with a positive parasitological test performed within 24 h of death and not included in the three above-mentioned situations were considered nonsevere active malarias. Finally, women showing malarial pigment in the liver, spleen, or placenta and no parasites were considered past infections.

All women testing positive for HIV PCR or Unigold test were considered HIV-positive. AIDS was defined by the 1993 CDC revised criteria [20].

### Statistical Methods

Data were analysed with SPSS version 11 and Epi Info 6.04d. Chi-square or Fisher exact test were used to evaluate differences in proportions between different groups. Relationships between the prevalence of each cause of death and age and parity were evaluated using a Chi-square test for trend.

Multinomial logistic regression analysis was done (Tables S1 and S2). Due to small numbers in some categories and therefore lack of statistical power it does not provide meaningful conclusions. This type of analysis could be done in future studies with larger sample sizes. *p*-Values less than 0.05 were considered significant.

## Results

### General Characteristics

There were 179 maternal deaths and 21,135 live births during the study period, resulting in a maternal mortality ratio of 8.47 per 1,000 live births. An autopsy was done in 151 (84.3%) of the deaths. In 28 maternal deaths the family refused to give permission for the autopsy, and another 12 were excluded because of inadequate sampling or absence of clinical information. Nonautopsied and incomplete maternal deaths were evenly distributed during the period of study. Thus, 139 (77.6%) autopsied women were included in this analysis. No differences in age were observed between women included and excluded because of missing information.

Demographic characteristics and the pregnancy status of the women are shown in Table 1. Mean age ± standard deviation was 26.1 ± 6.6 y, and mean parity was 2.8 ± 2.1 births.

**Table 1:**
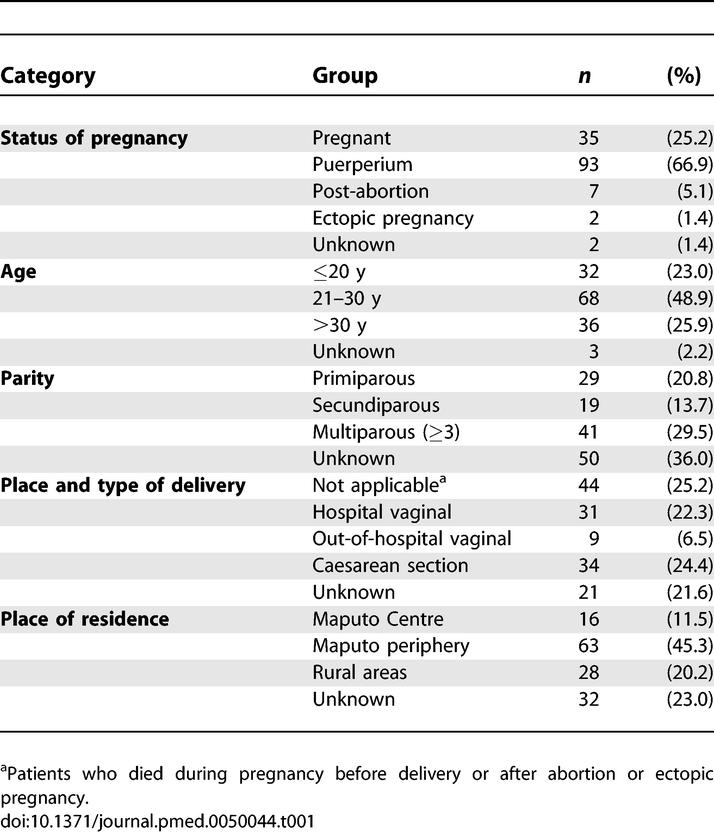
Demographic Characteristics of the Women in the Study

### Causes of Death

Table 2 shows the causes of death detected in the autopsies, and Table 3 shows the distribution of causes of death by age. The frequency of haemorrhage significantly increased with maternal age (Chi-squared [1 df] = 5.83; *p* = 0.02), whereas the frequency of puerperal septicaemia decreased with age (Chi-squared [1 df] = 5.57; *p* =0.02). The frequency of AIDS related conditions increased with age (Chi-squared [1 df] = 3.81; *p* = 0.05), while there was a non-significant trend for a reduced frequency of severe malaria (Chi-squared [1 df] = 3.08; *p* = 0.08) with increased maternal age (Table 3).

**Table 2:**
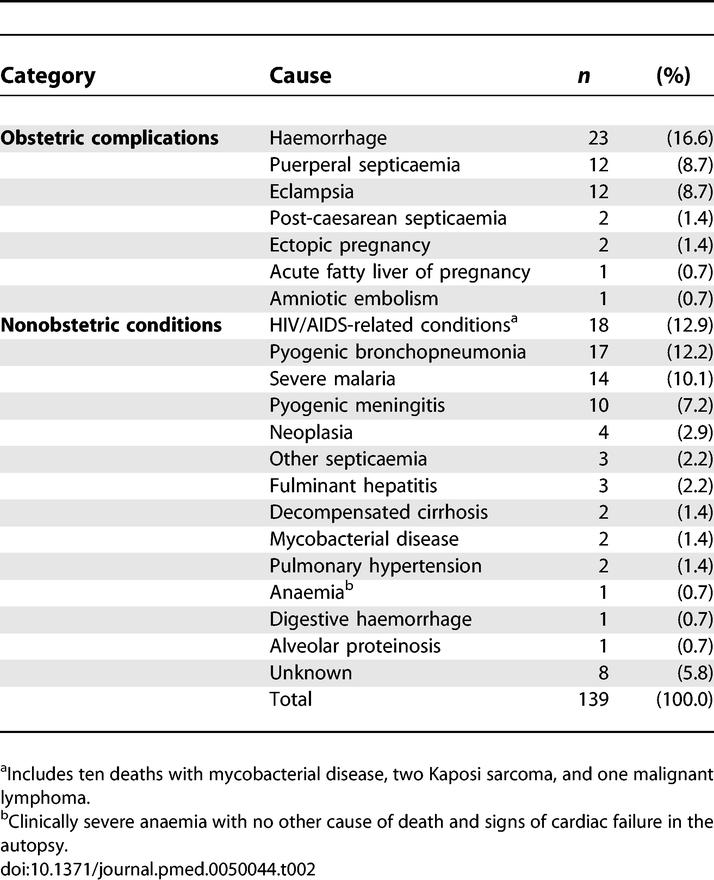
Causes of Death Found in the Autopsy

**Table 3:**
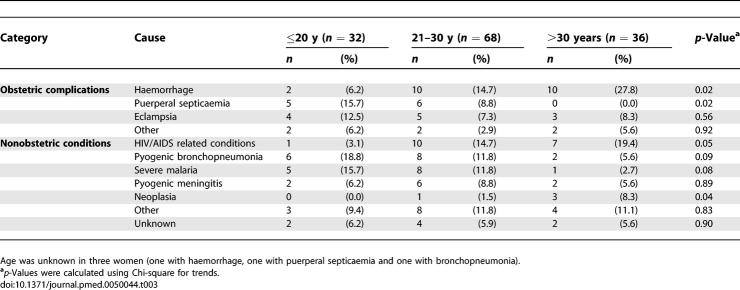
Distribution of the Causes of Death by Age

An HIV test was performed in 123 women, of whom 65 (52.8%) were positive. No material for HIV testing was available for 16 women. There was no statistical correlation between HIV infection and age: 34.5% of ≤ 20 y, 60.0% of 21–30 y, and 51.6% of > 30 y, were HIV positive, respectively (Chi-squared [1 df] = 0.07; *p* = 0.79).

#### Obstetric conditions.

Of those with known causes of death, 53 women (40.5%) died of obstetric complications (Table 2). Haemorrhage secondary to uterine atony, abruptio placentae, placenta accreta, placenta previa, or that related to uterine rupture due to obstructed labour, were the most frequent causes of death.

#### Nonobstetric conditions.

Of those with known causes, 78 (59.5%) women died of nonobstetric conditions (Table 2); of these, 67 (86% [i.e., 48.2% of all autopsied deaths]) died of infectious diseases.

HIV/AIDS-related complications were the most frequent nonobstetric cause of death. Ten AIDS patients had mycobacterial disease, one had mycobacterial pneumonia, and nine miliary mycobacteriosis, which in two women was associated with meningoencephalitis. Mycobacterium spp. were identified in Ziehl-Nielsen stains in all women. One woman had a multiple infection with miliary mycobacteriosis, pulmonary cryptococcosis, oral herpes simplex, and esophagic aspergillosis. Five pneumonias due to Pneumocystis jiroveci were identified. One of these had associated cerebral toxoplasmosis, and another cytomegalovirus pneumonia. Three patients had disseminated HIV/AIDS-associated neoplasms: two cases of Kaposi sarcoma, and a gamma-delta T cell hepato-splenic malignant lymphoma.

The distribution of the main causes of death in the HIV-positive and HIV-negative women is shown in Table 4. No differences were observed between HIV-positive and -negative women in the frequency of severe malaria (Table 4), or malaria infection (44/65 [67.7%] versus 40/58 [68.9%]; *p* = 1).

**Table 4:**
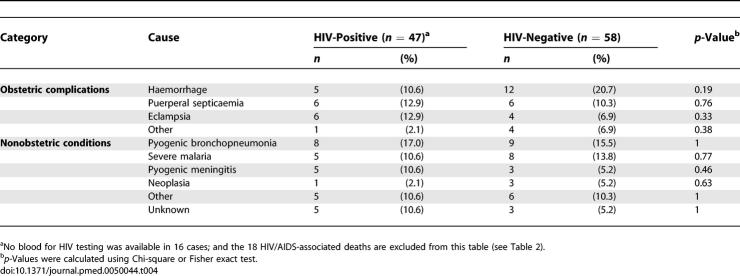
Autopsy Diagnoses in HIV-Positive and HIV-Negative Women

Seventeen women (12.2%), had pyogenic bronchopneumonia as a main cause of death. Severe bronchopneumonia was identified as a final event in two additional cases with eclampsia.

Fourteen women (10.1%) died of severe malaria. In five of them the diagnosis was based on the presence of abundant sequestered parasites in the CNS; in two women it was due to criterion (b), and in seven women it was due to criterion (c) (see Methods section).

Four neoplasms were identified: two large meningiomas with compressive phenomena in the CNS, one anaplastic oligodendroglioma, and one mesenteric fibromatosis with intestinal perforation.

Two miliary mycobacteriosis were detected in HIV-negative women. Three women with nonobstetric septicaemias were identified; one was secondary to a ruptured tubo-ovarian abscess, one to a dental abscess, and another to a perforation of a sigmoidal diverticulitis.

Malaria parasitaemia was found in 18/61 women (29.5%) tested before death. Histological evidence of either active or past malaria infection was observed in 92 (66.2%) of the 139 autopsies. The frequency of the causes of death in cases with and without histological evidence of active or past malaria infection is shown in Table 5. In the absence of severe malaria, none of the deaths were associated with having current or past malaria infection.

**Table 5:**
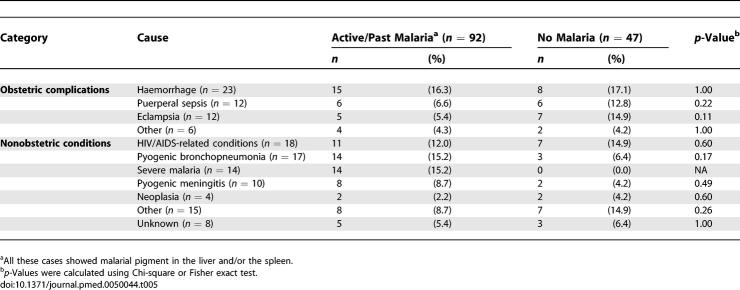
Causes of Death in Cases with and without Histological Evidence of Active/Past Malaria

Severe malaria was more frequent in women living in Maputo centre or periphery (2/16 [12.5%] and 9/63 [14.3%], respectively) than in women living in rural areas (1/28 [3.6%]) (*p* = 0.18). Evidence of active or past malaria was identified in 11/16 (68.7%) women living in Maputo centre, 44/63 women living in Maputo periphery (69.8%), and 23/28 (82.1%) women living in rural areas (*p* = 0.44).

Severe malaria was more frequent in primiparous women than in secundiparous and multiparous women (7/29 [24.1%]; 2/19 [10.5%], and 1/41 [2.4%], respectively, *p* < 0.01).

## Discussion

To our knowledge, this is the first description of the causes of maternal death in sub-Saharan Africa based on complete autopsies. In a previous study in the same setting no CNS examinations were conducted, thus preventing the accurate diagnosis of cerebral malaria [4,23]. Another study in Nigeria did not mention malaria specifically [24].

Obstetric complications accounted for 40.5% of maternal deaths for which a cause of death could be established. Haemorrhage represented the single most frequent cause. Puerperal septicaemia and eclampsia were also frequent causes of mortality. This result is in agreement with other studies performed in developing areas [2,4,9,25]. Improved management of the postpartum period that takes into account well-identified risk factors and provides improved facilities to correctly treat haemorrhages at the first care level could lead to a substantial reduction of maternal deaths.

Infectious diseases not directly related to the pregnancy accounted for 48.2% of the deaths. HIV/AIDS-related complications, pyogenic pneumonia, severe malaria, and pyogenic meningitis were responsible for over 40% of the maternal deaths. A striking finding was the high proportion of women dying of pyogenic pneumonia (21.8% of those dying of nonobstetric conditions), a condition of relatively low case-fatality rates in adults with no other clinical conditions (provided effective antibiotics are administered). Thus, prompt diagnosis and adequate treatment of infectious diseases may have a major impact on maternal mortality. HIV testing of pregnant and puerperal women with life-threatening conditions (regardless of whether or not an HIV test was done as part of routine antenatal care) is likely to reduce maternal mortality by improving clinician awareness of treatable conditions such as mycobacterial and pneumocystis infections. Mycobacterial disseminated infections were particularly common, and although mainly found in HIV-positive patients, they were also detected in a few HIV-negative women. A similar contribution of mycobacterial infections to maternal mortality has been reported in two studies on maternal mortality in Zambia and South Africa based on clinical records [25,26].

HIV infection had a major impact on maternal mortality. At least 12.9% of autopsied women died of diseases directly attributable to HIV infection. Moreover, we cannot exclude the possibility that some other infectious deaths could have been facilitated by HIV infection. More than half (52.8%) of the autopsied women had HIV antibodies. This prevalence is much higher than that reported for the adult population in Mozambique (16.2% as the average for the country and 20.7% for Maputo; unpublished data, Ministry of Health, Mozambique, 2004). As has been observed in other sub-Saharan countries, our results show that HIV is an important cause of maternal mortality in this region [27,28]. Interestingly, no deaths related to HIV infection were reported in Mozambique studies in the period 1989–1993 [4,23], indicating a rapid spread of the HIV epidemic in this region [29,30]. Alternatively, this observation might be explained by less frequent HIV testing in previous years. Thus, a substantial reduction in maternal mortality in sub-Saharan Africa would be achieved by increasing the uptake of HIV testing during pregnancy, antiretroviral treatment in HIV-positive pregnant women, and preventive measures in the general population.

We did not see an association with other diseases that have been reported to be more frequent and severe in HIV-positive pregnant women (Table 4), such as malaria [18]. This observation is probably due to the high prevalence of HIV in this series and the small sample size. Malaria was a direct cause of death in 10.1% (*n* = 14) of women, representing the fourth cause of death. No changes were found in the absolute number of deaths related to malaria compared with the period 1989–1993 [4,23]. This pattern suggests that the widely accepted assumption that maternal deaths directly attributable to malaria occur only in areas of unstable malaria transmission [31] needs to be revised. In our study, most deaths directly attributable to malaria occurred in women living in urban areas where malaria endemicity is low [19], indicating that the results for severe malaria may not be directly extrapolated to areas of high endemicity. However, the percentage of the population living in urban areas with low malaria transmission but surrounded by highly endemic areas is increasing in many developing countries. This change may lead to increased deaths due to severe malaria in people living in large cities. On the other hand, the observed increased risk of severe malaria in the lower age and parity groups, also found in a previous study in the same setting [23], is similar to the malaria pattern in pregnancy reported from areas of high transmission [16]. This observation suggests a previous acquisition of immunity against malaria, and therefore frequent exposure to the parasite.

A possible association between eclampsia and malaria has been reported as being more frequent in women with malaria-infected placentas [32,33]. However, we did not find any such association with malaria in our series, perhaps explained by the small number of women with eclampsia.

The maternal mortality ratio observed in our study (8.47 per 1,000 live births) is extremely high. This ratio has almost tripled during the last decade according to data reported from the same institution [4,23]. However, this observation has to be taken with caution since referral facilities have improved during this period, allowing more complicated deliveries to reach the hospital.

The main limitation of a study based in a tertiary referral hospital is that these results might not accurately reflect the wider community. The contribution of the different causes of death, as well as the magnitude of the maternal mortality ratio, may differ from those observed in rural areas, since complicated or high-risk pregnancies are referred to the hospital. On the other hand, the toll of obstetric haemorrhage in rural areas might be greater than that observed in this study, since it usually happens shortly after delivery with little time to reach a referral hospital. More careful clinical studies of serious illnesses in pregnant women, whenever possible accompanied by postmortem studies in patients with a fatal outcome (also in rural settings), are needed in developing countries to adequately guide health professionals and policy-makers in reducing this intolerable burden.

Infectious, treatable diseases accounted for a higher proportion of maternal deaths than did direct obstetric causes in this study. Thus, our findings show that there is a potential to greatly reduce maternal mortality in sub-Saharan Africa, not only through the improved performance of health services for obstetric complications, but also by implementing measures to adequately prevent and treat a specific group of infectious diseases.

## Supporting Information

Alternative Language Abstract S1 Translation of the Abstract into Spanish by Jaume Ordi (21 KB DOC)

Table S1 Regression Analysis: Multinomial Logistic Regression Estimates for Obstetric Complications (53 KB DOC)

Table S2 Regression Analysis: Multinomial Logistic Regression Estimates for Nonobstetric Conditions (55 KB DOC)
