# Supplementary material for: An Autopsy Study of Maternal Mortality in Mozambique: The Contribution of Infectious Diseases
Source: PLoS Med. 2008 Feb 19;5(2):e44. doi: 10.1371/journal.pmed.0050044 (PMC2245982; doi:10.1371/journal.pmed.0050044)
Supplement: Supplementary file 1 [file pmed.0050044.sd001.doc]

Un estudio autópsico sobre mortalidad materna en Mozambique: Cotribución de las enfermedades infecciosas.

###### RESUMEN

Fundamentos

La mortalidad maternal constituye un grave problema de salud que se concentra en zonas con bajos recursos. No existen datos fidedignos sobre sus causasque estén fundamentados en métodos rigurosos. Sin embargo, estos datos son necesarios para dirigir a tanto a los gestores como a los profesionales de la salud a reducir esta carga intolerable.

*Metodología*

Se ha llevado a cabo un estudio prospectivo entre Octubre de 2002 y Diciembre de 2004, sobre las causas de muerte materna en un hospital de tercer nivel en Maputo, Mozambique, realizando autopsias completas con estudio histológico. Se detectó VIH por métodos virológicos y serológicos. La malaria fue diagnosticada mediante examen histológico y parasitológico.

*Resultados*

En los 26 meses del estudio se produjeron 179 muertes maternas. Se realizó un estudio autópsico completo en 139 de ellas (77,6%), las cuales constituyeron la base del análisis. Sesenta y cinco (52,8%) de las mujeres analizadas fueron positivas para VIH. Las complicaciones obstétricas causaron el 38,2% de las muertes. Entre ellas la hemorragia fue la causa mas frecuente (16,6%). El 56,1% de las muertes fueron debidas a enfermedades no obstétricas. Entre ellas VIH/SIDA, bronconeumonía piógena, malaria grave y meningitis purulenta fueron las causas mas frecuentes (12,9%, 12,2%, 10,1% y 7,2% respectivamente). Se detectó infección micobacteriana como causa de muerte en 12 (8,6%) mujeres.

*Conclusiones*

En este hospital terciario de Mozambique, las enfermedades infecciosas causaron al menos la mitad de todas las muertes maternas, a pesar de que existen tratamientos disponibles para las tres causas mas frecuentes. Los reultados resaltan la necesidad de aplicar medidas de prevención efectivas y disponibles, tales como el tratamiento intermitente preventivo y mosquiteras impregnadas de insecticida para la malaria o vacunas para neumococo. Las muertes de causa obstétrica representan el fracaso de los sistemas de salud y requieren medidas urgentes de mejora.
