# Supplementary material for: An Autopsy Study of Maternal Mortality in Mozambique: The Contribution of Infectious Diseases
Source: PLoS Med. 2008 Feb 19;5(2):e44. doi: 10.1371/journal.pmed.0050044 (PMC2245982; doi:10.1371/journal.pmed.0050044)
Supplement: Supplementary file 2 [file pmed.0050044.st001.doc]

|  | Multinomial logistic regression estimates for obstetrical complications | | | | |  |
| --- | --- | --- | --- | --- | --- | --- |
|  | **Obstetrical complications** | | **RRR** | **[95% Conf. Interval]** | **p** |  |
|  | Puerperal septicaemia | |  |  |  |  |
|  |  | ≤20 years | 1 |  |  |  |
|  |  | 21-30 | 0.24 | 0.03 1.65 | 0.147 |  |
|  |  | > 30 years | 0.00* |  | 1.00 |  |
|  | Eclampsia | |  |  |  |  |
|  |  | ≤20 years | 1 |  |  |  |
|  |  | 21-30 | 0.25 | 0.03 1.86 | 0.176 |  |
|  |  | > 30 years | 0.15 | 0.02 1.26 | 0.081 |  |
|  | Other | |  |  |  |  |
|  |  | ≤20 years | 1 |  |  |  |
|  |  | 21-30 | 0.20 | 0.02 2.39 | 0.203 |  |
|  |  | > 30 years | 0.20 | 0.02 2.39 | 0.203 |  |
|  |  |  |  |  |  |  |
|  | Obstetrical Haemorrhage is the reference outcome | | | |  |  |
|  | * No events observed |  |  |  |  |  |
|  |  |  |  |  |  |  |
