# Supplementary material for: An Autopsy Study of Maternal Mortality in Mozambique: The Contribution of Infectious Diseases
Source: PLoS Med. 2008 Feb 19;5(2):e44. doi: 10.1371/journal.pmed.0050044 (PMC2245982; doi:10.1371/journal.pmed.0050044)
Supplement: Supplementary file 3 [file pmed.0050044.st002.doc]

|  | Multinomial logistic regression estimates for non obstetric conditions | | | | |  |
| --- | --- | --- | --- | --- | --- | --- |
|  | **Non obstetric conditions** | | **RRR** | **[95% Conf. Interval]** | **p** |  |
|  | Pyogenic bronchopneumonia | |  |  |  |  |
|  |  | ≤20 years | 1 |  |  |  |
|  |  | 21-30 | 0.13 | 0.01 1.35 | 0.088 |  |
|  |  | > 30 years | 0.05 | 0.00 0.66 | 0.024 |  |
|  | Severe malaria | |  |  |  |  |
|  |  | ≤20 years | 1 |  |  |  |
|  |  | 21-30 | 0.16 | 0.02 1.66 | 0.125 |  |
|  |  | > 30 years | 0.03 | 0.00 0.57 | 0.020 |  |
|  | Pyogenic Meningitis | |  |  |  |  |
|  |  | ≤20 years | 1 |  |  |  |
|  |  | 21-30 | 0.30 | 0.02 4.06 | 0.365 |  |
|  |  | > 30 years | 0.14 | 0.01 2.52 | 0.184 |  |
|  | Other | |  |  |  |  |
|  |  | ≤20 years | 1 |  |  |  |
|  |  | 21-30 | 0.27 | 0.02 3.08 | 0.290 |  |
|  |  | > 30 years | 0.19 | 0.01 2.50 | 0.207 |  |
|  |  |  |  |  |  |  |
|  | HIV/AIDS related conditions is the reference outcome | | | |  |  |
|  | Neoplasia is not evaluated because no events are observed among the reference age-group (≤20 years) | | | | | |
|  |  |  |  |  |  |  |
